# Grey parrots use inferential reasoning based on acoustic cues alone

**DOI:** 10.1098/rspb.2012.1292

**Published:** 2012-08-08

**Authors:** Christian Schloegl, Judith Schmidt, Markus Boeckle, Brigitte M. Weiß, Kurt Kotrschal

**Affiliations:** 1 Konrad Lorenz Forschungsstelle Gruenau, Core Facility University of Vienna, 4645 Grünau im Almtal, Austria; 2 Department of Behavioural Biology, University of Vienna, 1090 Vienna, Austria; 3 Department of Cognitive Biology, University of Vienna, 1090 Vienna, Austria; 4 Cognitive Ethology Laboratory, German Primate Center and Courant Research Centre for the Evolution of Social Behaviour, Georg August University of Göttingen, 37077 Göttingen, Germany; 5 ARGE Papageienschutz, 1090 Vienna, Austria; 6 Institute of Ecology and Evolution, Eberhard Karls University Tübingen, 72076 Tübingen, Germany

**Keywords:** grey parrots, reasoning, inference, shaking, cognition, convergence

## Abstract

Our ability to make logical inferences is considered as one of the cornerstones of human intelligence, fuelling investigations of reasoning abilities in non-human animals. Yet, the evidence to date is equivocal, with apes as the prime candidates to possess these skills. For instance, in a two-choice task, apes can identify the location of hidden food if it is indicated by a rattling noise caused by the shaking of a baited container. More importantly, they also use the absence of noise during the shaking of the empty container to infer that this container is not baited. However, since the inaugural report of apes solving this task, to the best of our knowledge, no comparable evidence could be found in any other tested species such as monkeys and dogs. Here, we report the first successful and instantaneous solution of the shaking task through logical inference by a non-ape species, the African grey parrot. Surprisingly, the performance of the birds was sensitive to the shaking movement: they were successful with containers shaken horizontally, but not with vertical shaking resembling parrot head-bobbing. Thus, grey parrots seem to possess ape-like cross-modal reasoning skills, but their reliance on these abilities is influenced by low-level interferences.

## 1. Introduction

Reasoning is among the cornerstones that define human cognitive abilities. As such, it has gained increasing interest in comparative cognition, but numerous studies suggest that human-like reasoning skills are limited in non-human animals [1]. In one of the most commonly used paradigms, the so-called inferential reasoning by exclusion task [2], animals have to find a reward hidden in one of two containers [2–8]. Before they make their choice, the subjects are informed about the content of one of the containers; in the inference condition, the animals *see* that one container is empty. A variety of species solves this task and chooses the alternative, non-empty container [2–8]. However, the assumption that their success is based on the logical inference that the reward must be in the alternative container has been criticized [1,9], as it appears more parsimonious that the subjects simply avoided the empty container. In line with this critique, most non-human species have difficulties when the presence and absence of the reward is not directly perceived. This is the case, for instance, when the occurrence of noise during the shaking of a container indicates the location of the food, and the unbaited container is noiselessly shaken in the corresponding reasoning condition. Here, the subjects have to make a two-step deduction. First, the presence and absence of noise has to be connected to the presence or absence of the reward, respectively. This information must then be used to deduce that the *absence* of noise in one container is predicting the *presence* of the reward in the other container [4,9]. In this task, only the great apes were instantaneously successful [4] and managed to use the absence of noise to infer that the reward is located in the alternative container. By comparison, in three different studies, capuchin monkeys (*Cebus apella*) made the same inference either only after elaborate training [6], relied on the occurrence only, but not the lack of noise [10], or failed entirely [5]. Olive baboons (*Papio hamadryas anubis*) neither used the presence nor the absence of a noise cue [7], whereas dogs (*Canis familiaris*) relied on noise even if the cue was not causally relevant (a mobile phone ringing in a non-shaken container) [11]. However, the dogs also failed to respond appropriately to the absence of noise. Three-year-old human children can solve this task, even though only 4-year-olds may reach adult-like performance levels and are equally successful when relying on the presence and the absence of the rattling noise [12].

Non-human primates repeatedly performed poorly in other acoustic problem-solving tasks as well, even if they had mastered visual versions of the same task [13]. It was thus suggested that primates generally may not be overly attentive to acoustic cues [14], whereas the same may not be true for other cognitively advanced species. To investigate this possibility, we tested six captive African grey parrots (*Psittacus erithacus*) in the acoustic inferential reasoning task. Grey parrots are renowned for their sophisticated cognitive skills [15–21] and solved reasoning tasks in other domains [15,22]; because of their phylogenetic distance to the primates, they serve as a prime example for convergent cognitive evolution in mammals and birds [23].

## 2. Experiment 1: shaking of the containers

### (a) Methods

#### (i) Subjects

Subjects were six, from 10 to 35 years old African grey parrots (three males) of not fully known individual history, housed in a parrot rescue centre in Vienna, Austria. Previously, the birds had participated in the visual version of the same experiment (J. Schmidt, K. Kotrschal & C. Schloegl 2009, unpublished data), in which the containers were lifted to reveal the content (see also [3,8] for studies using the same procedure); two subjects had also participated in a string-pulling task (C. Schloegl & J. Schmidt 2008/2009, unpublished data). The birds were housed together with other, non-tested birds in an indoor–outdoor aviary (each section 3 × 6 m, height indoor: 3 m, height outdoor up to 4 m). The testing compartment (1.2 × 0.7 m) was situated within the indoor aviary, but visually isolated from the other birds. Participation in the experiments was voluntary, and the subjects were free to leave the compartment any time.

#### (ii) Procedure

We used two opaque plastic containers (height: 6 cm, diameter: 7.5 cm) that were placed on square plastic cards (8.5 × 8.5 cm) to allow the lifting of the containers while being turned upside down. Plastic cards were covered with cloth to avoid unintended noises caused by the reward when lifting the containers. The containers and the bird were situated on a wooden platform (0.6 × 0.35 m and 1.3 m above ground). For each trial, the experimenter baited the containers out of view of the bird and held and/or shook the containers approximately 15 cm in front of the platform. Then, the containers were placed on the platform simultaneously, equidistantly to and on both sides of the bird. The experimenter stepped back and stood behind the platform at a distance of approximately 30 cm, looking straight ahead, remaining motionless until the bird had made its choice. The location of the baited container (left/right) was semi-randomized, with the stipulation that the reward was not positioned on the same side for more than two consecutive trials. To make a choice, the bird approached a container and turned it over with its beak. As a reward, we used pieces of walnuts, a highly preferred food item not regularly available outside of testing.

Each session began with two warm-up trials, in which one piece of food was hidden in full view of the birds in one of the containers. The birds chose the baited container consistently and never chose incorrectly in both warm-up trials. These warm-up trials were directly followed by up to 12 test trials (i.e. one test session). In one case, a bird left the testing compartment during the session and did not return; the session was terminated and continued on the next day. Within each session, we presented three trials of each of the following four conditions:

both: the experimenter lifted and shook both containers simultaneously for about 3 s;
baited: the experimenter lifted both containers, but shook the baited one only;
empty: as before, but the empty container was shaken; and
control: the experimenter lifted both containers, but did not shake them. After 3 s, she returned the containers to the platform.

To investigate whether the birds were influenced by the type of shaking movement, the containers were shaken vertically for six sessions, and horizontally shaken for another six sessions; four birds received vertical shaking first and two birds horizontal shaking first. Vertical shaking consisted of rapid and repeated up-and-down movements. Horizontal shaking was similar, but containers were moved parallel to the platform and in a 90° angle from the birds.

We created a unique and random sequence in which the conditions were to be presented for each session and each bird was tested with the same random sequence. As the order in which conditions were presented for the first time may be of relevance, we here report the sequence in the first trials of the first session of experiment 1 (and the retest after experience training, see later text), until each of the four conditions had occurred for the first time (i.e. the first six trials): ‘both’—‘control’—‘both’—‘empty’—‘empty’—‘baited’.

After the initial task consisting of 12 sessions, we administered a training procedure to those birds that did not significantly prefer the correct cup in the ‘baited’ as well as in the ‘both’ condition. Training consisted of two sessions of 12 trials each. At the start of a trial, the experimenter positioned an empty container on the platform and tilted the container towards the bird to show that it was empty. The experimenter then shook this container horizontally without noise occurring. In the next step, the experimenter took a food reward and showed it to the bird. She then dropped the reward into the container and shook it so that a rattling noise was clearly audible. Finally, the bird was allowed to retrieve the food. After the two training sessions, these birds were retested in the first task, but with horizontal shaking only.

#### (iii) Analysis

For logistical reasons, video-recording was possible only from the beginning of experiment 3 (see below), and choices were scored live by the experimenter (J.S.). Choices were unequivocal in all cases. Group performance against the hypothetical chance level of 50 per cent was tested using one-sample *t*-tests. As *t*-tests are robust against violations of normal distribution, we used this procedure also with not fully normalized data [24]. To assess individual performances, we used binomial tests.

To investigate which parameters influenced choice behaviour, we constructed generalized linear mixed models (GLMMs) with ‘choice’ (correct/wrong) as binomial response variate and subject identity as random term. Shaking movement (horizontal and vertical), condition (both, baited, empty, control), the session number per shaking treatment (1–6), the running number of each trial within a session (1–12), the order of presentation (vertical or horizontal shaking first) and movement × condition and movement × session interactions were entered as fixed terms. According to standard stepwise model reduction procedures, we sequentially deleted fixed terms in order of decreasing significance, whereby the least significant term was determined after each removal step [25,26]. Deletion of fixed terms continued until only terms with a significance value below 0.1 remained. This was then considered the final model. Excluded terms were re-entered one by one into the final model to confirm that they did not explain a significant part of the variation. For each factor remaining in the final model, we calculated pairwise post hoc comparisons using the sequential Sidak–Holmes procedure; terms were only regarded as being significant if *p* < 0.05. To evaluate the relative importance of terms in the final model, we compared their effect sizes [26], whereby the effect size of a factor was considered to be the range of effect sizes (minimum to maximum) across the factor levels. To allow comparisons of the accuracies of the full and the final model, we present each model's corrected Akaike's information criterion (AIC). AICs quantify the relative fit of each model, whereby lower values indicate a better fit [27]. All tests were conducted two-tailed using SPSS 19, with *α* = 0.05.

### (b) Results

**Figure 1.**
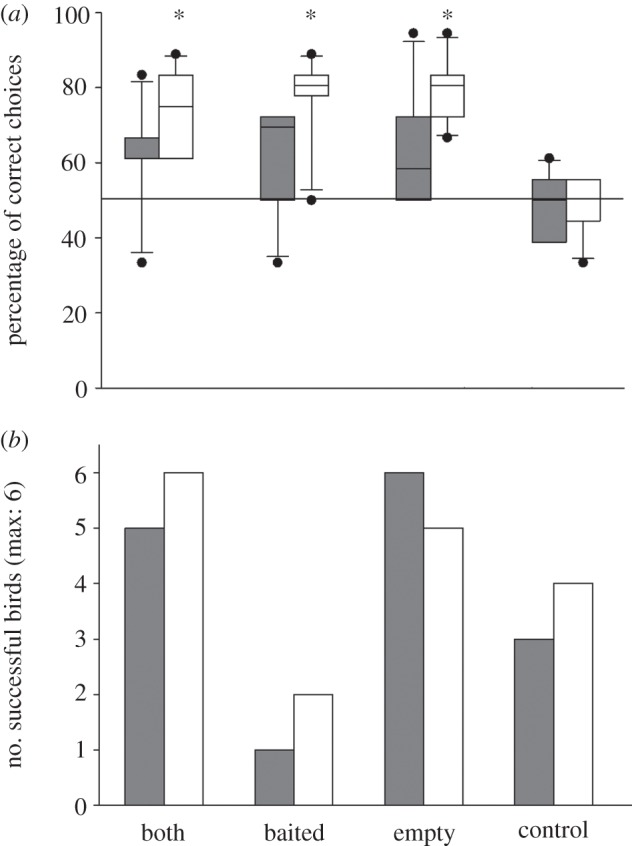
(*a*) Performance of six African grey parrots in the three test conditions and the control condition of the first experiment (vertical (filled grey bars) versus horizontal (open bars) treatment). The horizontal line indicates the 50% chance level. Asterisks above the bars indicate significant deviation from chance level (one-sample *t*-test). Boxplots show median and 25th and 75th percentiles, whiskers show 10th and 90th percentiles and dots represent outliers. (*b*) Number of successful subjects in the first experiment on the first trial of each condition in each treatment.

The birds were significantly more successful in the three test conditions (both, baited, empty) than in the control condition (GLMM, see table 1*a* for the full and the final model, Sidak: all *p* ≤ 0.001), whereas no difference could be found between the test conditions (Sidak: all *p* ≥ 0.719). Thus, similar to the great apes and in contrast to all other species tested so far [4–7,10,28], the grey parrots spontaneously used the presence *and the absence* of a rattling noise to deduce the location of hidden food. Interestingly however, their performance was strongly influenced by the direction of the shaking movement: In the ‘baited’ and in the ‘empty’ condition, the birds were significantly more successful when the containers were shaken horizontally than when they were shaken vertically (Sidak: *p* = 0.011 and *p* = 0.009, respectively). Their performance in the ‘both’ condition was also better when the containers were shaken horizontally than when shaken vertically, but the comparison failed to reach significance (Sidak: *p* = 0.076). In the control condition, in which the containers were not shaken and thus perceptually identical for both treatments (horizontal versus vertical), we did not find a significant difference between horizontal and vertical control treatment (Sidak: *p* = 0.681; figure 1*a*). Accordingly, the birds correctly identified the baited container in all three test conditions when the containers were shaken horizontally (one-sample *t*-tests, all *p* ≤ 0.005), but not in the corresponding control condition or in any of the four conditions when the containers were shaken vertically (all *p* ≥ 0.104; figure 1*a*). More subjects significantly preferred the baited container in the ‘baited’, ‘both’ and ‘empty’ condition when the containers were shaken horizontally (five, four and four individuals, respectively), than when the containers were shaken vertically (one parrot in the ‘both’ and in the ‘empty’ condition; all binomial tests: *p* ≤ 0.031). The birds’ performance was independent from the sequence in which they were confronted with the two shaking movements (table 1*a*).

**Table 1.** (*a*) GLMM test statistics of the first experiment. (*b*) GLMM test statistics of the replication of the first experiment after the shake/rotate task; in both cases, correct choice was entered as a binomial choice variable; subjects were entered as random effects. We provide effect sizes for the final model only (see electronic supplementary material for details).

fixed terms	d.f.	*F*	*p*	*F*	effect size	*p*
(*a*)	full model (AIC: 3823.176)			final model (AIC: 3812.9)		
shaking movement	1	3.710	0.054	10.918	0.754	0.001
condition	3	11.302	<0.001	11.293	0.603	<0.001
order of movements	1	0.111	0.739			
session number	1	0.202	0.653			
trial number	1	5.241	0.022	5.238	−0.049	0.022
movement × condition	3	2.177	0.089	2.176	0.912	0.089
movement × session	1	0.237	0.627			
(*b*)	full model (AIC: 1970.185)			final model (AIC: 1964.08)		
condition	3	9.866	<0.001	9.848	0.965	<0.001
session number	1	1.686	0.195			
trial number	1	7.930	0.005	7.971	−0.089	0.005

The subjects’ performance remained relatively constant over the course of the experiment. A very moderate decline in later trials within sessions (effect size: −0.049; table 1*a*) was detectable, but we did not find any evidence for a change of performance across sessions (table 1*a*). This argues against a rapid learning process during the course of the experiment. The near-perfect first-trial performance in the ‘both’ and in the ‘empty’ condition with both shaking movements (figure 1*b*) adds additional support to this interpretation. It is noteworthy that the birds apparently managed to continue on this high success level with the horizontal shaking movement only; nevertheless, this finding needs to be treated with caution because of the small number of subjects. Interestingly, the subjects performed relatively poorly in the first trial of the ‘baited’ condition (figure 1*b*).

Despite their large overall success, we found considerable inter-individual performance differences. All subjects performed significantly above chance level in at least one test condition (binomial test), but only three of the six birds reliably chose the container producing noise in the ‘both’ *and* in the ‘baited’ condition (see electronic supplementary material, table S1 for individual performance data). This may be attributed to the simultaneous shaking of both containers in the ‘both’ condition, which may have increased attentional demands. Therefore, those three birds that had not been individually significant in the ‘both’ *and* ‘baited’ condition received two training sessions. When afterwards being retested in the shaking task (using horizontal shaking only), these three birds selected the baited container significantly above chance in the ‘both’ and in the ‘baited’ condition (binomial-test, all *p* ≤ 0.031), but failed to reach significance in the ‘empty’ condition, with success rates of 55.6 per cent, 66.7 per cent and 72.2 per cent of the trials, respectively (see also electronic supplementary material, table S2).

## 3. Experiment 2: rotate versus shake

The first experiment demonstrated that grey parrots can use the presence and the absence of noise to identify the baited container; however, it is not clear whether they indeed came to understand the causal link between shaking, occurrence of noise and the presence and absence of food. For instance, it was suggested that the success in the ‘empty’ condition may have resulted from the avoidance of a container being shaken noiselessly [4,28]. We investigated this possibility in the next experiment. The subjects had a choice between a noiseless container shaken horizontally and a noiseless container being rotated three times around its horizontal axis. The logic here is that if the birds had solved the ‘empty’ condition by avoiding the combination of horizontal shaking and lack of noise, they should continue to do so in the current experiment. However, they should choose at random if they can assess that the silence during horizontal shaking is not indicative for a reward because of the noiseless movement of both containers.

### (a) Methods

The subjects and the general procedure were identical to the first experiment, with the exception that we did not apply warm-up trials. We conducted four sessions with horizontal shaking, consisting of 12 trials each. We presented three conditions per session, with four trials per condition in randomized order:

both: both containers were shaken simultaneously (identical to experiment 1);
control: both containers were lifted but not shaken (identical to experiment 1); and
rotate versus shake: both containers were lifted and shaken simultaneously. One container was shaken horizontally (as before), the other container was rotated three times around its horizontal axis. In this condition, both containers were un-baited.

### (b) Results

As in the previous experiment, the birds preferred the baited container in the ‘both’ (*p* < 0.001), but not in the ‘control’ condition (*p* = 0.793). However, we found no preference for any movement in the ‘rotate versus shake’ condition (see also electronic supplementary material, table S3), neither over the course of the entire experiment (one-sample *t*-test: *p* = 0.576), nor in the very first trial (two of six birds chose the rotated container). The rotated container was also not chosen more often in the first half of the trials than in the second half of the trials (paired *t*-test: *p* = 0.576). Thus, this experiment illustrates that the ‘empty’ condition in the first experiment was not solved through an intrinsic avoidance of a horizontally shaken noiseless container.

## 4. Experiment 3: retest of experiment 1 with horizontal shaking only

We next replicated our original shaking experiment, but restricted it to the horizontal shaking movement. This was carried out to test whether the exposure to a condition in which no food could be obtained, i.e. the ‘rotate versus shake’ condition, had an effect on the birds’ performance.

### (a) Methods

Subjects, procedure and analysis were identical to experiment 1 with the exception that horizontal shaking was used only. One session was interrupted, because the bird left the testing compartment and did not return; it was continued on the next day. A second coder analysed 20 per cent of the trials and the interobserver reliability was excellent (Cohen's *κ* = 0.93).

### (b) Results

**Figure 2.**
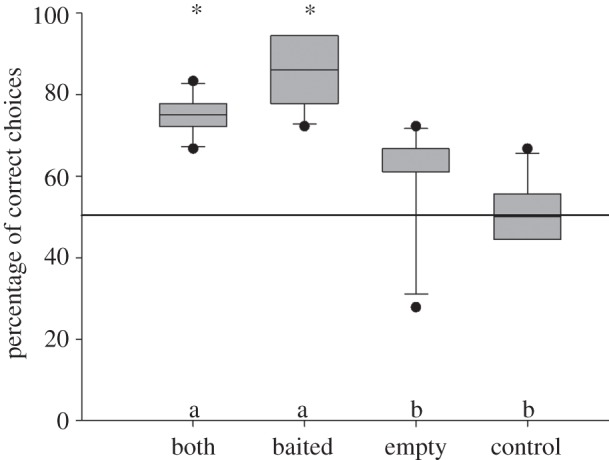
Performance of the birds in experiment 3, the replication of the shaking task (conducted after the ‘shake-rotate’—control). Asterisks above the bars indicate a significant deviation from chance level (one-sample *t*-test). Different letters above the *x*-axis indicate significant differences between the conditions (post hoc Sidak test). Boxplots show median, and 25th and 75th percentiles, whiskers show 10th and 90th percentiles and dots represent outliers.

The birds again preferred the baited cup significantly more often in the ‘both’ and in the ‘baited’ condition than in the ‘control’ condition (Sidak: *p* ≤ 0.003; see table 1*b* for full and final model). However, we could no longer find a difference between the ‘empty’ and the ‘control’ condition (Sidak: *p* = 0.38), and the birds’ choices differed from chance only in the ‘both’ (one-sample *t*-test: *p* < 0.001) and the ‘baited’ condition (*p* < 0.001), but not in the ‘empty’ (*p* = 0.248) and the ‘control’ condition (*p* = 0.611). Still, five of the six birds chose the correct container in the ‘empty’ condition preferentially, even though the strength of their preferences decreased from the first experiment (see electronic supplementary material, table S4 for individual performance data). The lack of significance on the group level is largely attributable to a single subject that switched its preference and now selected the incorrect container in the majority of trials (figure 2).

## 5. Experiment 4: playback

The previous experiment confirmed that grey parrots rely on the occurrence of rattling noise to identify the baited container. However, it is still possible that they may have learned to associate noise with a reward, but did not understand the causal link between the two. Thus, in our last experiment we conducted playback trials, in which the containers were not shaken, but the noise was broadcasted from small speakers attached to the experimenter's wrist and hidden under her sleeves.

### (a) Methods

The same birds served as subjects, and the general procedure was identical to experiment 1. In a total of six sessions, three birds left the testing compartment before the end of a session (each bird did so twice); these sessions were continued on the following day.

The birds received 16 sessions with horizontal shaking, and we presented two warm-up and 12 test trials per session. The test trials were distributed across six different conditions:

baited: both containers were lifted and the baited one was shaken (identical to experiment 1); and
empty: both containers were lifted and the empty one was shaken (identical to experiment 1).

These two conditions were presented four times per session.

As unrewarded probe trials we presented once per session:

shaking, noise playback: both containers were lifted, one container was shaken and noise was played back on the side of the shaken container;
no shaking, noise playback: as before, but the experimenter lifted the containers without shaking them; playback of noise randomly from the left or the right speaker;
shaking, silent playback: as ‘shaking, noise playback’, but a recording containing silence only was played back from the side of the shaken container; and
no shaking, silent playback: as ‘no shaking, noise playback’, but with a recording of silence.

During all trials, the experimenter wore two loudspeakers (X-mini capsule speakers; 5 × 5 × 4.5 cm) attached to her wrist, hidden in the sleeves of her shirt (see electronic supplementary material, figure S1). The loudspeakers were connected to a MP3 player (Samsung Digital Audio YP-U2R). In contrast to other playback controls [4,6], this allowed us to broadcast the sound of rattling food from close to each of the two containers without changing the visual appearance of the set-up.

For the playback, we used six different recordings of noise produced by the shaking movement of walnut pieces. Recordings were obtained with a Marantz PMD 660 solid-state recorder using a Sennheiser K6ME67 microphone, and were saved as *.wav files (sampling rate = 48 kHz, amplitude resolution = 16 bit). We further produced six synthetical files containing no noise to control for hints the experimenter might have given during the playback procedure, e.g. the switch-on click. Mono playback files and silent noise files were produced with Adobe Soundbooth CS4 for Macintosh and were played back at naturally occurring sound pressure levels of 64 dB (at 1 m distance). We randomly picked one file from the pool of recordings for each trial. A second observer coded 5 per cent of the trials and the interobserver reliability was excellent (Cohen's *κ* = 1).

### (b) Results

**Figure 3.**
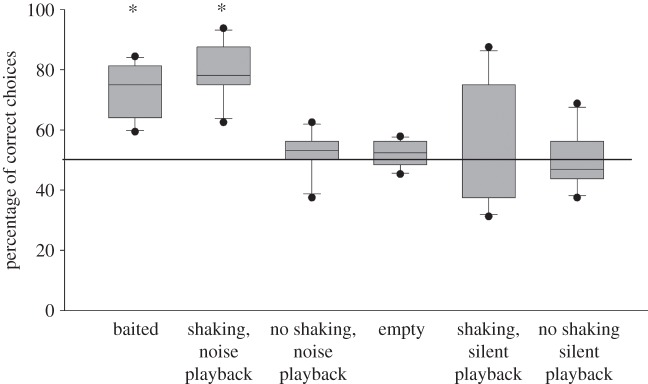
Performance of six grey parrots in the playback experiment (experiment 4). Asterisks above the bars indicate a significant deviation from chance level (one-sample *t*-test). Boxplots show median and 25th and 75th percentiles, whiskers show 10th and 90th percentiles and dots represent outliers.

When a rattling noise was audible and the container was shaken, the birds significantly preferred the shaken container. Importantly, this was the case regardless of whether the noise was produced by the food (‘baited’ condition) or by the playback (‘shaking, noise playback’; one-sample *t*-test: in both cases, *p* ≤ 0.002). On an individual level, five birds significantly preferred the correct container in the ‘baited’ condition, and three did so if the container was shaken and the noise was played back (‘shaking, noise playback’; binomial-tests, all *p* ≤ 0.033; see electronic supplementary material, table S5 for individual performance data). The birds, however, did not have a preference for the ‘noisy’ container if the noise stemmed from the playback but the container was not shaken (one-sample *t*-test, *p* = 0.576). In the ‘empty’ condition and in both conditions in which a silent playback was broadcasted, the birds did not have a significant preference for a container (all *p* ≥ 0.378; figure 3). Only one bird preferred the non-shaken container, if the other container was shaken and a silent playback was broadcasted (‘shaking, silent playback’), whereas none of the birds had a preference in any of the other conditions. The reliance on the noise in the ‘baited’ and in the ‘shaking, noise playback’ condition suggests that the birds treated the noise as being caused by the food, even if it was played back. In consequence, the lack of preference for the noisy container in the ‘no shaking, noise playback’ condition thus cannot be explained as an artefact caused by using recorded noise. Instead, it is more plausible that the birds did not prefer the noisy container, because the causal chain was broken and the noise occurred without shaking movement.

## 6. General discussion

Through a series of experiments we found compelling evidence for the ability of African grey parrots to use noise created during the shaking of containers to detect hidden food. Even from the very first trial, our subjects could also use the absence of noise in a shaken container to infer that food must be in the other, non-shaken container. Such behaviour has so far been shown only in the great apes [4,28] but not in any other non-human animal [5–7,11]. Human children solve this task from an age of 3–4 years, and the birds’ success rate was comparable to those of the 3-year-olds [12]. The parrots’ near-perfect first-trial performances as well as the results of our control experiments suggest that an understanding of the causal properties underlying the task is the most likely explanation for the birds’ performance. Nevertheless, we cannot entirely exclude an explanation based on associative learning of a complex rule [1]. The birds may have learned within their very first trial that a compound contingency of shaking and noise jointly indicates the presence of the reward. Still, causal understanding and learning are most likely tightly connected and should not be treated as two opposing or discrete mechanisms. Instead, it appears more realistic to consider causal understanding and learning processes as working in concert. Thereby, a ‘folk-physical’ understanding of the world's contingencies is adjusted through experience [29].

One of the most striking results is the large effect of the direction of the shaking movement. One potential explanation is an interference with pre-defined grey parrot behavioural cues. We noted that during vertical shaking trials, some birds turned around or began with head bobbing, a typical parrot action pattern featuring a repeated up-and-down movement of the head that is displayed in various contexts. Even though speculative, the perceptual similarity of head bobbing and vertical shaking might have distracted the birds and elicited alternative responses. This resembles observations that certain behavioural responses cannot be elicited through classical conditioning, if the anticipated response interferes with a component of the species’ behavioural repertoire [30].

It is remarkable that these largely experimentally naive parrots solved a task that experimentally highly experienced monkeys were not able to solve [5,6]. However, this does not necessarily imply that parrots outperform primates cognitively, as these tasks may fit better to the sensory orientation of parrots [31]. It was suggested that primates may not be good at solving discriminatory tasks in the acoustic domain [13] owing to the relative unimportance of acoustic information in their feeding ecology [14]. Capuchin monkeys, however, do tap on stones and use acoustic feedback to assess the stones’ suitability as pounding tools [32]. Hence, noise cues clearly play a role for capuchins, but the absence of noise may be irrelevant to them. For the moment it remains unclear why grey parrots make use of this information, but the birds’ behaviour is consistent with that of other studies highlighting the inferential abilities of this species. For instance, Alex, Irene Pepperberg's language-trained grey parrot, made inferential choices in a vocal labelling task [22], and another grey parrot has recently been shown to deduce the actions of a human experimenter in a food hiding and pilfering task [15]. A critic may argue that the birds may have picked up on subtle experimenter-given cues. This, however, appears extremely unlikely, because the parrots solved the task only when the cups were shaken horizontally and stopped choosing correctly in the ‘empty’ condition of experiment 3 which we did not anticipate. In consequence, if they had been guided by experimenter-given cues, the birds should have chosen the baited container also in these trials.

Interestingly, on their very first trials of experiment 1, our parrots chose correctly in the ‘empty’ and the ‘both’, but not in the ‘baited’ condition. This finding came as a surprise, but might be explained by the sequence of trial presentations. The first ‘baited’ trial was conducted in direct succession of two consecutive ‘empty’ trials, which initially may have confused the birds; however, they apparently overcame this confusion rapidly and reliably chose the correct container in all test conditions. From there on, they were consistent in their reliance on the presence of the noise, whereas they stopped responding to the absence of the noise after the shake–rotate task (experiment 2). This may be explained by a partial extinction because of a repeated use of unrewarded probe trials, and on the first glance seems to contradict the idea of causal understanding. However, silence is a more ambiguous cue than the presence of noise. Under physically salient conditions, the occurrence of noise during a shaking movement is causally predictive for the presence of something, whereas the lack of noise is predicting the presence of something under certain arbitrary rules only, such as those employed in the first experiment (i.e. ‘food is hidden in one of two locations’). However, this became partially invalid when we introduced the unrewarded ‘shake-rotate’-trials. Thus, with this step we changed the arbitrary contingencies of the experiment, whereas the underlying causal rules remained intact.

Taken together, our findings demonstrate for the first time that a non-ape species is able to solve an auditory ‘inference by exclusion’ task instantaneously. The strong first-trial performance as well as the performance in the control tasks suggest that the parrots may indeed be capable of causal reasoning, which is in line with findings obtained in another reasoning task [15]. Yet, their performance is error-prone and may be influenced by interferences through stimulus–response processes.
